# A Robust Dual-Microphone Generalized Sidelobe Canceller Using a Bone-Conduction Sensor for Speech Enhancement

**DOI:** 10.3390/s21051878

**Published:** 2021-03-08

**Authors:** Yi Zhou, Haiping Wang, Yijing Chu, Hongqing Liu

**Affiliations:** 1 School of Communication and Information Engineering, Chongqing University of Posts and Telecommunications, Chongqing 400065, China; zhouy@cqupt.edu.cn (Y.Z.); s180131236@stu.cqupt.edu.cn (H.W.); liuhongqing@cqupt.edu.cn (H.L.); 2 State Key Laboratory of Subtropical Building Science, South China University of Technology, Guangzhou 510641, China

**Keywords:** generalized sidelobe canceller, speech enhancement, bone-conduction sensor, voice activity detection

## Abstract

The use of multiple spatially distributed microphones allows performing spatial filtering along with conventional temporal filtering, which can better reject the interference signals, leading to an overall improvement of the speech quality. In this paper, we propose a novel dual-microphone generalized sidelobe canceller (GSC) algorithm assisted by a bone-conduction (BC) sensor for speech enhancement, which is named BC-assisted GSC (BCA-GSC) algorithm. The BC sensor is relatively insensitive to the ambient noise compared to the conventional air-conduction (AC) microphone. Hence, BC speech can be analyzed to generate very accurate voice activity detection (VAD), even in a high noise environment. The proposed algorithm incorporates the VAD information obtained by the BC speech into the adaptive blocking matrix (ABM) and adaptive noise canceller (ANC) in GSC. By using VAD to control ABM and combining VAD with signal-to-interference ratio (SIR) to control ANC, the proposed method could suppress interferences and improve the overall performance of GSC significantly. It is verified by experiments that the proposed GSC system not only improves speech quality remarkably but also boosts speech intelligibility.

## 1. Introduction

Speech technology plays an important role in speech communication and human-computer interaction. Microphone arrays have been widely studied in speech enhancement because of their great performance in enhancing the quality and intelligibility of the received speech signal [1]. They are capable of sound source localization [2], which is essential for beamformers and indoor location [3,4]. The generalized sidelobe canceller (GSC) is an effective technique for an adaptive microphone array, which is commonly used in speech enhancement applications. The conventional GSC contains three parts: the fixed beamformer (FBF), the blocking matrix (BM), and the adaptive noise canceller (ANC). The issue is that the conventional BM does not retain noise well and even suffers from desired signal leakage, which limits the noise reduction performance of the GSC. Usually, the adaptive blocking matrix (ABM) is preferred to extract noise and reject desired signals. The control of coefficients update for ABM and ANC is crucial to the final performance, which has been studied by many researchers. In [5], a control method was designed by utilizing signal-to-interference ratio (SIR) estimation obtained with the output powers of FBF and ABM. Hoshuyama et al. proposed a GSC with a new ABM using coefficient-constrained adaptive filter and an ANC with norm-constrained adaptive filter [6]. Herbordt and Kellermann [7] implemented a similar GSC in the frequency domain. Later, Yoon, Tashev, and Malvar [8] incorporated the sound-source presence probability estimated from the instantaneous direction of arrival of the input signals and voice activity detection (VAD) into the ABM. Khayer et al. proposed replacing the blocking matrix in GSC with a linear constrained minimum variance (LCMV) beamformer to alleviate the leakage of the desired signal and effectively reduce the noise [9]. Li et al. extended the direction of arrival (DOA) estimation to the traditional GSC module, which enhanced the blocking effect of the blocking matrix and reduced the leakage of the desired signal [10].

**Figure 1:**
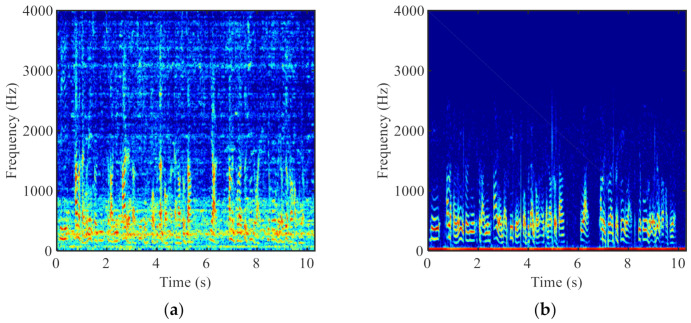
Spectrograms of (**a**) the AC and (**b**) the BC speech signals.

Despite the effectiveness of the various proposed methods, the accurate control of the ABM and ANC, especially under highly non-stationary noise and low signal-to-noise ratio (SNR) conditions, is still very challenging. To improve the control accuracy, other information offered by new types of sensors can be a complement to the microphone signals. Various sensors have been widely used, especially in the Internet of Things [11,12]. Among them, the non-acoustic, bone-conduction (BC) sensor is a promising selection for speech enhancement applications. Unlike the air-conduction (AC) microphone, the BC sensor is comparatively less sensitive to the environmental acoustic noise since it senses the vibration of sounds through bones of the skull [13]. Figure 1 illustrates the spectrograms of the AC and BC speech signals that were recorded simultaneously in the same noisy environment. It can be observed the BC speech signal is much less deteriorated by the ambient acoustic noise, but its high frequency spectrum (>800 Hz) is seriously attenuated due to the low-pass nature of the human body. This leads to the poor intelligibility of the BC speech signal, which hinders its direct use.

There are two categories of approaches employing BC speech signals for speech enhancement. The first one is to explore the non-linear mapping of BC speech signals to AC speech signals [14,15,16,17]. Recently, a deep neural network was used to map the spectral coefficients of the linear prediction coding of BC speech to the coefficients of AC speech [18]. Liu et al. utilized a deep noise reduction autoencoder to achieve the abovementioned mapping [19]. The second is to utilize the characteristics of BC speech signal to assist the AC speech enhancement, for example, those based on the VAD estimation [20], on the low frequency substitution [21], and on the a priori SNR estimation [22].

The dual-microphone array has advantages of low cost, small size, and ultra-low power consumption and has been widely used in wearable devices such as hearing aids, earphones, and smart glasses, in which BC sensors are suitably embedded. Therefore, this paper focuses on the dual-microphone array framework, where we propose a novel robust dual-microphone GSC assisted by BC sensor. With the accurate VAD consistently obtained through the BC signal even in low SNR environments, the successful control of the ABM can be achieved.

By further incorporating the VAD information together with the SIR information into the control of ANC, a satisfactory result can also be obtained. The effectiveness of the proposed GSC algorithm is then confirmed by experiments in the presence of the non-stationary and diffuse noises.

The rest of the paper is organized as follows: Section 2 introduces the conventional GSC structure. Section 3 elaborates the details of the proposed algorithm. Simulation experiments and results are presented and discussed in Section 4. Conclusions are given in Section 5.

## 2. Previous Work

**Figure 2:**
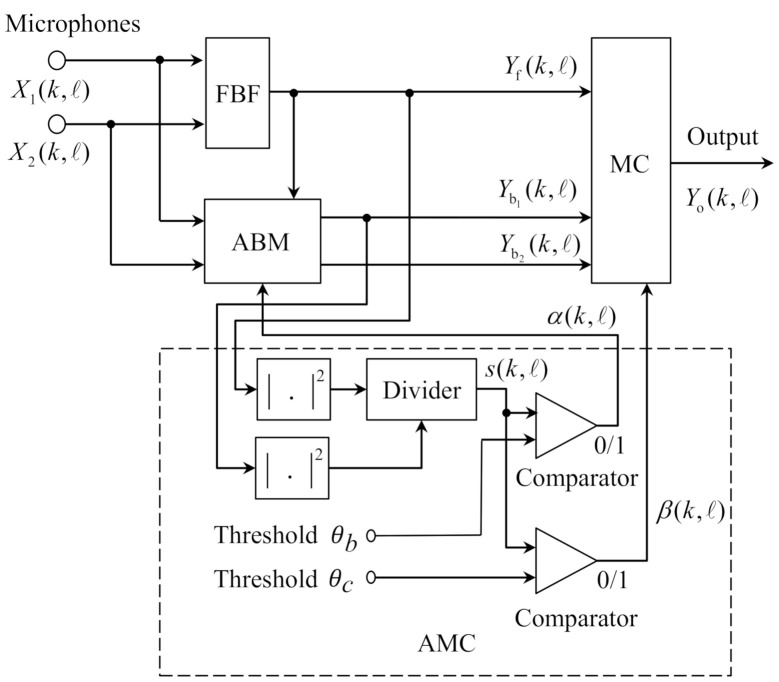
Structure of the conventional GSC proposed in [5].

The work in this paper is based on the GSC structure with ABM that was proposed in [5]. As shown in Figure 2, for a dual-microphone array, the GSC is composed of a FBF, an adaptation-mode controller (AMC), an ABM, and a multiple-input canceller (MC). Let k and ℓ denote the frequency bin and the frame indices, respectively.

First, the two microphone inputs $X_{i}(k,\ell )$ (*i* = 1, 2) enter the FBF that can steer the main beam to the direction of desired signal. $Y_{f}(k,\ell )$ is the output of the FBF and is used as the reference signal for the ABM. The ABM subtracts the desired signal from each channel input $X_{i}(k,\ell )$ to produce the reference noise signal $Y_{b_{i}}(k,\ell )$ for the MC. $Y_{b_{i}}(k,\ell )$ ideally contains only the noise components. On the contrary, MC adaptively subtracts the noise signal from $Y_{f}(k,\ell )$ to obtain the desired signal. The coefficients in the ABM and the MC are updated by the normalized least mean square (NLMS) algorithm that is controlled by AMC. The AMC consists of two power estimators, one divider, and two comparators [5]. In Figure 2, s(k,ℓ) is the smoothed power ratio of the FBF output signal $Y_{f}(k,\ell )$ to an ABM output signal $Y_{b_{i}}(k,\ell )$. The coefficients of the ABM are updated when s(k,ℓ) is larger than the threshold $\theta_{b}$, while the adaptation of the MC is performed when s(k,ℓ) is smaller than threshold $\theta_{c}$. The adaptive filtering algorithm for the ABM is implemented as follows:

(1) $$Y_{b_{i}}(k,\ell )=X_{i}(k,\ell )-W_{b_{i}}(k,\ell )Y_{f}(k,\ell ),$$

(2) $$W_{b_{i}}(k,\ell +1)=W_{b_{i}}(k,\ell )+\alpha (k,\ell )\mu_{b}(k,\ell )Y_{f}{}^{*}(k,\ell )Y_{b_{i}}(k,\ell )$$

where * denotes just the complex conjugate, $W_{b_{i}}(k,\ell )$ is the coefficients of the ABM, and the adaptation switch α(k,ℓ) for the ABM is controlled as follows:

(3) $$\alpha (k,\ell )=\left\{\begin{array}{ll} 1 & \mathrm{if}\;s(k,\ell )>\theta_{b}\\ 0 & \mathrm{otherwise} \end{array}\right.,$$

(4) $$s(k,\ell )=\frac{p_{f}(k,\ell )}{p_{b}(k,\ell )},$$

(5) $$p_{f}(k,\ell )=\gamma p_{f}(k,\ell -1)+(1-\gamma ){\left|Y_{f}(k,\ell )\right|}^{2},$$

(6) $$p_{b}(k,\ell )=\gamma p_{b}(k,\ell -1)+(1-\gamma ){\left|Y_{b_{i}}(k,\ell )\right|}^{2},$$

where $p_{f}(k,\ell )$ is a power estimate of $Y_{f}(k,\ell )$, $p_{b}(k,\ell )$ is a power estimate of $Y_{b_{i}}(k,\ell )$, and γ is a smoothing factor satisfying 0≤γ≤1. The normalized step size $\mu_{b}(k,\ell )$ at the ℓ-th frame is:

(7) $$\mu_{b}(k,\ell )=\mu_{1}{\left[\theta_{b}+{\tilde{S}}_{f}(k,\ell )\right]}^{-1}$$

where $\mu_{1}$ is a fixed step size, $\theta_{b}$ is a small number to avoid $\mu_{b}(k,\ell )$ from becoming too large, and ${\tilde{S}}_{f}(k,\ell )$ is the smoothed power estimation of the FBF output, given by:

(8) $${\tilde{S}}_{f}(k,\ell )=\varphi_{b}{\tilde{S}}_{f}(k,\ell -1)+(1-\varphi_{b}){\left|Y_{f}(k,\ell )\right|}^{2}$$

where $\varphi_{b}$ is a parameter that is used to control the update speed.

The adaptation of the MC is obtained as:

(9) $$Y_{o_{i}}(k,\ell )=Y_{f}(k,\ell )-W_{a_{i}}(k,\ell )Y_{b_{i}}(k,\ell ),$$

(10) $$W_{a_{i}}(k,\ell +1)=W_{a_{i}}(k,\ell )+\beta (k,\ell )\mu_{a}(k,\ell )Y_{b_{i}}{}^{*}(k,\ell )Y_{o_{i}}(k,\ell )$$

where $W_{a_{i}}(k,\ell )$ is the coefficients of the MC, $\mu_{a}(k,\ell )$ is the step size that is similar to $\mu_{b}(k,\ell )$, and the adaptation switch β(k,ℓ) for the MC is controlled by:

(11) $$\beta (k,\ell )=\left\{\begin{array}{ll} 0 & \mathrm{if}\;s(k,\ell )>\theta_{c}\\ 1 & \mathrm{otherwise} \end{array}\right..$$

The index s(k,ℓ) is treated as an estimate of the SIR in that the main component at the FBF output is the desired signal and the main component at the ABM output is the interference. In that sense, s(k,ℓ) is explored to distinguish between desired signal and interference with the purpose of correct coefficients update in the ABM and MC.

Although the idea of the above GSC algorithm is very practical, it still has some drawbacks. First, if the performance of ABM in a certain frame is unsatisfactory, the coefficient update decision of all future frames of ABM and MC will probably be inaccurate, which leads to an overall poor performance. In addition, the estimation of the SIR is inaccurate in a strong noise environment. These problems are addressed using BC speech to control ABM and MC in the next section.

## 3. Proposed Robust GSC

### 3.1. System Overview

**Figure 3:**
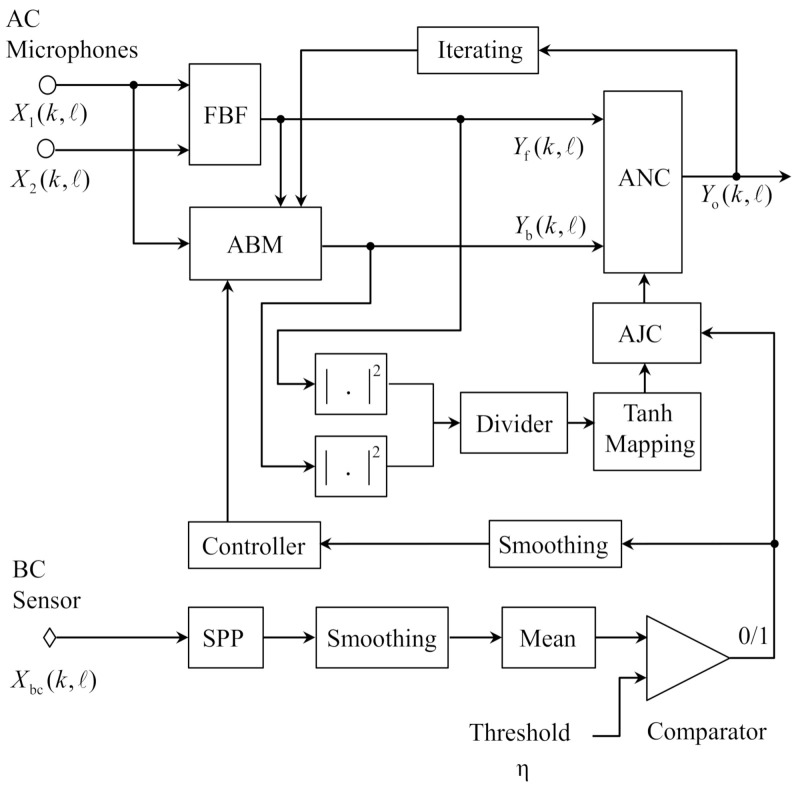
Schematic diagram of the proposed BCA-GSC algorithm.

The structure of the proposed robust GSC is depicted in Figure 3, where the AJC means the adaptive joint controller. The crucial part of the algorithm is to obtain the VAD information by estimating the speech presence probability (SPP) of the BC speech signal. The first microphone is designated as the reference microphone and the well-known robust super-directive beamformer [23] is used as the FBF. The output of the FBF and the first microphone signal are used for ABM, which is controlled by the VAD information obtained through the BC speech signal. Then the outputs of the FBF and ABM are sent to ANC, which is jointly controlled by the VAD information and the SIR acquired by the output powers of the FBF and ABM. The adaptations in the ABM and ANC need classification, due to the contrary relationships between the desired signal and the noise for the adaptation algorithm. For the adaptation algorithm in the ABM, the noises are the reserved objects and the desired signal is the object of blocking. In the ANC, however, the desired signal is the retained object and the noises are the objects to be eliminated. Therefore, the coefficients in the ABM should be updated in the speech presence components, while the coefficients update should be performed in the speech absence components in the ANC. To further improve the performance, the final output of the GSC is fed into the ABM as the reference signal, and the outputs of the FBF and ABM are sent back to ANC. This iteration is performed only once to obtain the final enhanced speech.

### 3.2. VAD Based on BC Sensor

In the proposed algorithm, the VAD information is obtained via the BC speech signal. The primary task is to estimate the SPP in each frame of the BC speech signal. In [24], the a posteriori SPP based on minimum mean square error (MMSE) criterion is given by:

(12) $$p(k,\ell )=\left[\right.1+(1+\xi )e^{-\;\frac{{\left|Y(k,\ell )\right|}^{2}}{{\hat{\sigma }}_{v}{}^{2}(k,\ell -1)}\;\frac{\xi }{1+\xi }}{\left.\right]}^{-1}$$

where ξ denotes the *a priori* SNR and is a fixed value of $10\log_{10}(\xi )=15$ dB [24] for reducing the overestimated spectral noise power and computational complexity, ${\left|Y(k,\ell )\right|}^{2}$ denotes the power of the BC speech signal, and ${\hat{\sigma }}_{v}^{2}(k,\ell -1)$ denotes the noise power estimate of the previous frame signal.

The noise power estimation is updated with the SPP-based noise estimation algorithm, as follows:

(13) $${\hat{\sigma }}_{v}{}^{2}(k,\ell )=\alpha_{v}(k,\ell ){\hat{\sigma }}_{v}{}^{2}(k,\ell -1)+(1-\alpha_{v}(k,\ell )){\left|Y(k,\ell )\right|}^{2}$$

where the time-frequency dependent smoothing factor:

(14) $$\alpha_{v}(k,\ell )=\alpha_{\min}+(1-\alpha_{\min})p(k,\ell )$$

is utilized to control the update rate. $\alpha_{\min}$ is a constant satisfying $0\leq \alpha_{\min}\leq 1$. If the noise power estimate ${\hat{\sigma }}_{v}^{2}(k,\ell )$ underestimates the true noise power $\sigma_{v}^{2}(k,\ell )$, the *a posteriori* SPP in (12) will be overestimated. It follows that then the noise power will not be tracked as quickly as expected. In the extreme case, when ${\hat{\sigma }}_{v}^{2}(k,\ell )$ seriously underestimates the true noise power $\sigma_{v}^{2}(k,\ell )$, the a posteriori SPP is close to 1, p(k,ℓ)=1. Then the noise power will no longer be updated, even though ${\left|Y(k,\ell )\right|}^{2}$ may be small with respect to the true noise power $\sigma_{v}^{2}(k,\ell )$.

To avoid a stagnation of the noise power update owing to an underestimated noise power, additional mechanisms are further employed. First, the inter-frame smoothing on p(k,ℓ) is performed, as:

(15) $$\tilde{p}(k,\ell )=\alpha_{p}\tilde{p}(k,\ell -1)+(1-\alpha_{p})p(k,\ell )$$

where $\alpha_{p}$ is a smoothing factor satisfying $0\leq \alpha_{p}\leq 1$, and the initial value of $\tilde{p}(k,\ell )$ is set to 0.5. Then, if the smoothed *a posteriori* SPP $\tilde{p}(k,\ell )$ is larger than 0.99, it can be considered that the update may have stagnated, and the current a posteriori SPP estimate p(k,ℓ) will be forced to be less than 0.99, as:

(16) $$p(k,\ell )=\left\{\begin{array}{ll} \min(0.99,\;p(k,\ell )), & \mathrm{if}\;\tilde{p}(k,\ell )>0.99\\ p(k,\ell ), & \mathrm{otherwise} \end{array}.\right.$$

In the proposed algorithm, only the specific part of the BC speech signal whose frequency spectrum lies between 70 Hz and 800 Hz is used for VAD because no human voice is below 70 Hz and the power of BC speech is attenuated significantly above 800 Hz. Note that for female voices or general higher pitch voices, the upper limit of the applicable BC speech frequency can be up to 1.5 kHz, and setting the upper frequency limit to 800 Hz can also obtain accurate VAD information. Let $p_{m}(\ell )$ represent the average of the smoothed a posteriori SPP $\tilde{p}(k,\ell )$ in those frequency bins that satisfy the above conditions in the ℓ-th frame. The decision criterion of VAD now is:

(17) $$I(\ell )=\left\{\begin{array}{cc} 1, & \mathrm{if}\;p_{m}(\ell )>\eta \\ 0, & \mathrm{otherwise} \end{array}\right.$$

where I(ℓ)=1 represents the speech presence, and I(ℓ)=0 represents speech absence. η is a threshold satisfying 0<η<1.

**Figure 4:**
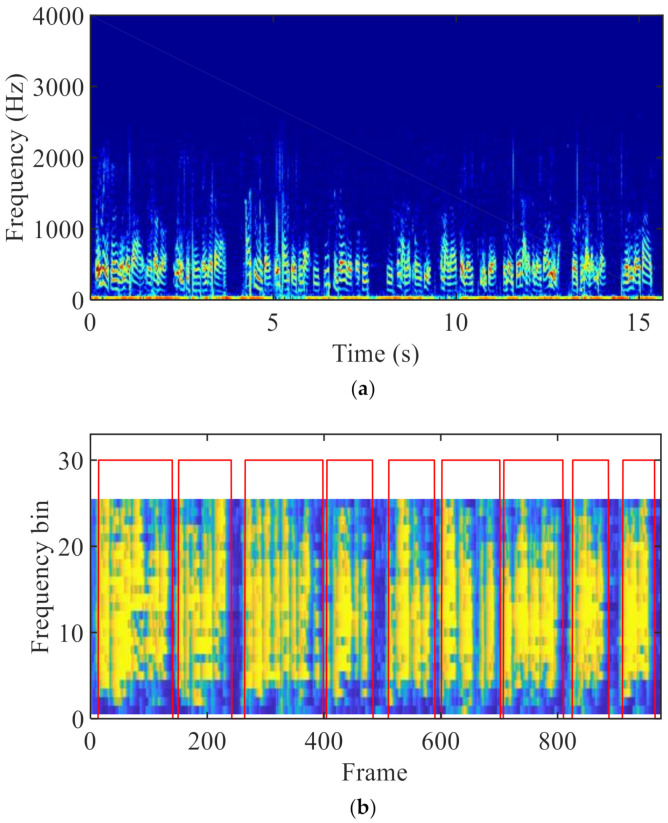
(**a**) Spectrogram of BC speech and (**b**) SPP of BC speech and smoothed VAD.

Note that VADs for the ABM and ANC are different. This is because the VAD in the ABM should make all the speech presence frames be detected at the expense of some speech absence frames being misjudged. However, the speech absence frames need be detected as much as possible in the ANC. Therefore, a smoothing operation is performed when using VAD to control the ABM. Specifically, I(ℓ) jumps from 0 to 1 only if $t_{1}$ speech presence frames appear successively; while I(ℓ) switches from 1 to 0 once $t_{2}$ speech absence frames appear successively. This paper employs $I_{s}(\ell )$ to denote the smoothed I(ℓ). However, VAD for the ANC does not need to be smoothed. Experimental results showed that this approach outperforms the method where both the ABM and the ANC use the smoothed VAD. Figure 4 shows the spectrogram of a BC speech and the VAD result obtained. In Figure 4b, the shade of yellow represents the a posteriori SPP $\tilde{p}(k,\ell )$, and the red rectangles denote the result of the VAD. Note that the VAD result is originally 0 or 1, and for the convenience of observation, the amplitude of VAD is matched with SPP graph and then plotted with SPP in a graph. It can be seen that the estimation of the SPP is not delayed, and the result of the VAD is accurate.

### 3.3. Improved ABM

The ABM is a spatial rejection filter, where it rejects the desired signal and passes the noise. The FBF output $Y_{f}(k,\ell )$ is sent to the ABM as a reference signal for the latter. The received signal $X_{1}(k,\ell )$ from the first microphone is used as the input signal of the ABM. The ABM adaptively subtracts the components from the $X_{1}(k,\ell )$ that are correlated to $Y_{f}(k,\ell )$. That is:

(18) $$Y_{b}(k,\ell )=X_{1}(k,\ell )-W_{b}(k,\ell )Y_{f}(k,\ell )$$

where $Y_{b}(k,\ell )$ is the ABM output, and ideally it contains only the interference signals. $W_{b}(k,\ell )$ is the coefficients of the ABM.

The proposed algorithm utilizes the VAD information $I_{s}(\ell )$ obtained from the BC speech signal to control the update of the adaptive filter $W_{b}(k,\ell )$ of length *L*. In this paper, the recursive least squares (RLS) algorithm [25] is employed to update the adaptive coefficients due to the efficiency in terms of convergence speed. These coefficients can be calculated by solving the following linear problem in a recursive way:

(19) $$\left(R_{f}(k,\ell )+\kappa I)W_{b}(k,\ell +1)=P_{f}(k,\ell \right)$$

where the covariance matrix at the ℓ-th frame $R_{f}(k,\ell )$, estimated by using the forgetting factor (FF) λ as well as the recursive weight $\lambda_{\ell -i}(\ell )=\lambda \lambda_{\ell -i-1}(\ell -1)$ with $\lambda_{0}(\ell )=1$, is:

(20) $$R_{f}(k,\ell )=\sum_{i=L}^{\ell }\lambda_{\ell -i}(\ell )Y_{f}(k,\ell )Y_{f}^{T}(k,\ell )$$

and the cross-correlation vector is:

(21) $$P_{f}^{}(k,\ell )=\sum_{i=L}^{\ell }\lambda_{\ell -i}(\ell )X_{1}(k,\ell )Y_{f}(k,\ell ).$$

***I*** and κ in (19) are, respectively, the identity matrix of size *L* and a positive parameter that prevent the RLS algorithm from divergence when the covariance matrix is ill conditioned. Equation (19) can be updated using the QR decomposition technique, when and only when the time dependent control factor, as shown below, is 1:

(22) $$\xi_{b}(\ell )=\left\{\begin{array}{c} 1,\\ \mathrm{if}\;I_{s}(\ell )==1\\ 0,\\ \mathrm{otherwise} \end{array}\right..$$

The QRD implementation can be found in Table 1 [25,26], where the input signal is replaced by $Y_{f}(k,\ell )$ while the desired signal is replaced by $X_{1}(k,\ell )$. Under these settings, the value of w(n) gives the ABM coefficients $W_{b}(k,\ell )$.

From Table 1, it can be seen that the rank-one update of the covariance matrix $R_{f}(k,\ell )$ can be implemented by updating the Cholesky factor $R_{}^{\left(1\right)}(n)$ of $R_{f}(k,\ell )$ recursively (1st QRD in recursion (i), Table 1). Note, the FF λ can be made variable to better track the parameters in a time-varying environment. The QRD is executed once for the data vector and once for the regularization $\left[\sqrt{\kappa }z_{m},0\right]$ at each time instant, where $z_{m}$ is the m-th row of the identity matrix I of size L. The computational complexity of solving (19) is identical to that of the conventional QRRLS algorithms, which is o(L^2^). Note, we have used italic and bold letters to denote matrices and vectors in Table 1 to show that the QRRLS implementation can be applied to both ABM and ANC. The iteration number n updates only when the switch (22) (or (27) in the next subsection) is on.

### 3.4. Improved ANC

The goal of the ANC is to reject the noise and extract the desired signal. It eliminates the portions in the FBF output $Y_{f}(k,\ell )$ that are correlated to the ABM output $Y_{b}(k,\ell )$. That is:

(23) $$Y_{o}(k,\ell )=Y_{f}(k,\ell )-W_{a}(k,\ell )Y_{b}(k,\ell )$$

where $Y_{o}(k,\ell )$ is the ANC output and $W_{a}(k,\ell )$ is the weight coefficients of the ANC. In the proposed algorithm, the update of $W_{a}(k,\ell )$ is jointly controlled by the SIR and the VAD information I(ℓ). The method of obtaining the SIR s(k,ℓ) is the same as (4), whereas the proposed algorithm does not compare the obtained SIR with a threshold to obtain a binary result like (11), but rather maps the SIR to 0-1 using the tanh function as:

(24) $$C_{0}(k,\ell )=\tanh(s(k,\ell ))=\frac{e^{s(k,\ell )}-e^{-s(k,\ell )}}{e^{s(k,\ell )}+e^{-s(k,\ell )}},$$

(25) $$C(k,\ell )=\left\{\begin{array}{ll} 1, & \mathrm{if}\;\;C_{0}(k,\ell )>\lambda_{1}\\ 0, & \mathrm{if}\;\;C_{0}(k,\ell )<\lambda_{0}\\ C_{0}(k,\ell ), & \mathrm{otherwise} \end{array}\right.$$

where C(k,ℓ) is a parameter to control the coefficients update of the ANC, $\lambda_{1}$ and $\lambda_{0}$ are two thresholds.

The strategy of AJC is that $W_{a}(k,\ell )$ is also updated by RLS when the VAD result indicates non-speech frame, otherwise C(k,ℓ) is utilized to control the update speed of RLS for better removing noise that leaked into speech frames. In particular:

(26) $$\left(R_{b}(k,\ell )+\kappa I)W_{a}(k,\ell +1)=P_{b}(k,\ell \right)$$

where $R_{b}(k,\ell )$ and $P_{b}(k,\ell )$ are, respectively, the covariance matrix of the input $Y_{b}(k,\ell )$ and cross-correlation vector between $Y_{f}(k,\ell )$ and $Y_{o}(k,\ell )$ as defined in (20) and (21). The FF, however, is variable according to the parameter $\xi_{a}(k,\ell )$ that controls the update speed, given by:

(27) $$\xi_{a}(k,\ell )=\left\{\begin{array}{cc} 1-C(k,\ell ), & \;\;\;\;\;\mathrm{if}\;\;I(\ell )\;==1\\ 1, & \;\mathrm{otherwise} \end{array}\right..$$

The variable FF can be computed from:

(28) $$\lambda (k,\ell )=1-\mu_{a}(k,\ell )$$

where:

(29) $$\mu_{a}(k,\ell )=\xi_{a}(k,\ell )\mu_{2},$$

and $\mu_{2}$ is a small positive constant that acts as a fixed step-size.

Equation (26) with a variable FF can also be implemented by using the QR decomposition as shown in Table 1. The input is replaced by $Y_{b}(k,\ell )$ while the desired signal is replaced by $Y_{o}(k,\ell )$. Under these settings, the adaptive filter w(n) gives the value of $W_{a}(k,\ell )$.

The tanh function in (24) is a monotonically increasing function. When current frame is detected as the speech presence frame, it can be seen from (24) and (27) that the larger s(k,ℓ) and the smaller $\xi_{a}(k,\ell )$ are produced, which leads to slower update of $W_{a}(k,\ell )$, and vice versa. The role of (25) is to stop parameter updating at strong SIR values and to speed up parameter updating at weak SIR values.

### 3.5. Iteration

The output of the ANC should contain less noise than the output of the FBF. Theoretically, letting ANC output $Y_{o}(k,\ell )$ instead of the FBF output $Y_{f}(k,\ell )$ be the reference signal of ABM can reserve more noise in the output of ABM, which leads to improved noise reduction performance of ANC. In this case, the ABM adaptively subtracts the components from the $X_{1}(k,\ell )$ that are correlated to $Y_{o}(k,\ell )$, as:

(30) $$Y_{b}(k,\ell )=X_{1}(k,\ell )-W_{b}(k,\ell )Y_{o}(k,\ell ).$$

The outputs of the FBF and ABM are still sent to the ANC as same as (23). The control method of coefficients update of the ABM and ANC is the same as above. The experimental results show that iterating only once can lead to a better performance than no iteration while iterating multiple times produces no further performance improvements. In this work, only one iteration is adopted.

## 4. Experimental Results

**Figure 5:**
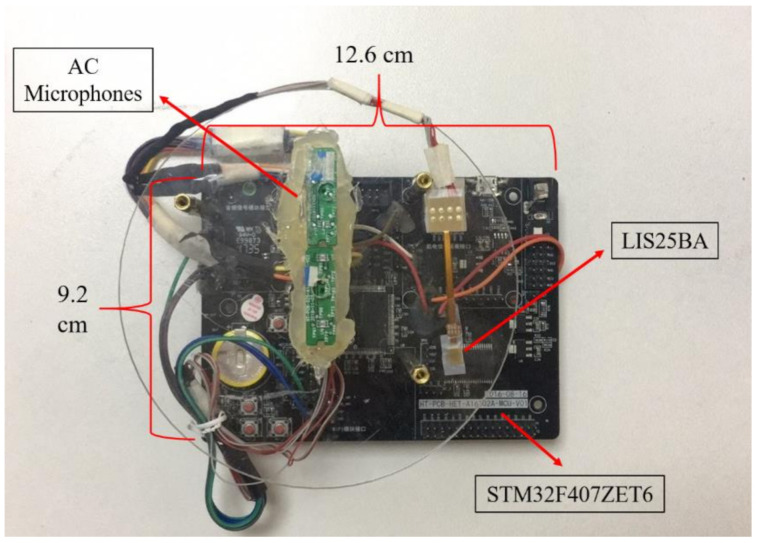
Collection equipment of AC and BC speech signals.

To validate the usefulness of the proposed BCA-GSC algorithm, we compare its performance with the conventional GSC algorithm [5] in various noise environments. The performance evaluation includes objective quality and intelligibility measures. Sensors are usually used as terminal equipment to acquire information [27,28]. In this paper, the STM32F407ZET6 development board (STMicroelectronics, Geneva, Switzerland) equipped with two AC microphones and a LIS25BA bone vibration sensor (STMicroelectronics, Geneva, Switzerland) was employed to collect speech signals, as shown in Figure 5. The AC microphones used are InvenSense T3902 (TDK InvenSense, Sunnyvale, CA, USA). Their package size is 3.5 × 2.65 × 0.98 mm, the SNR is 64.5 dB, and the power consumption in the ultra-low power mode is as low as 185 µA. The LIS25BA enjoys the advantages of low cost, low power consumption and high sensitivity and so on. Note that the device that can collect BC speech is not the only one. To simulate a wearable device application, the distance between the two AC microphones was set to 3 cm. The sampling frequencies of the AC speech and the BC speech were 16 kHz and 8 kHz, respectively. A Hanning window with 50% overlap was used for AC speech (512 samples) and for BC speech (256 samples). The 512-point and 256-point FFT were performed on the AC speech and the BC speech, respectively, which ensures the same frame number. Due to the conjugate symmetry of the Fourier transform, only 257 frequency bins and 129 frequency bins were used per frame for AC and BC speech respectively, which include both the direct current (DC) frequency component and the Nyquist frequency component. Other parameters used in the algorithm were as follows: VAD: $\alpha_{\min}=0.8$, $\alpha_{p}=0.8$, η=0.3, $t_{1}=3$, and $t_{2}=5$. ANC: $\lambda_{1}=0.8$, $\lambda_{0}=0.1$, and $\mu_{2}=0.08$.

**Figure 6:**
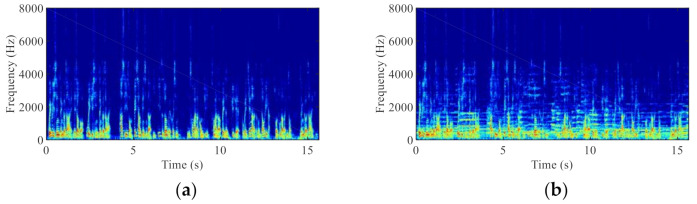

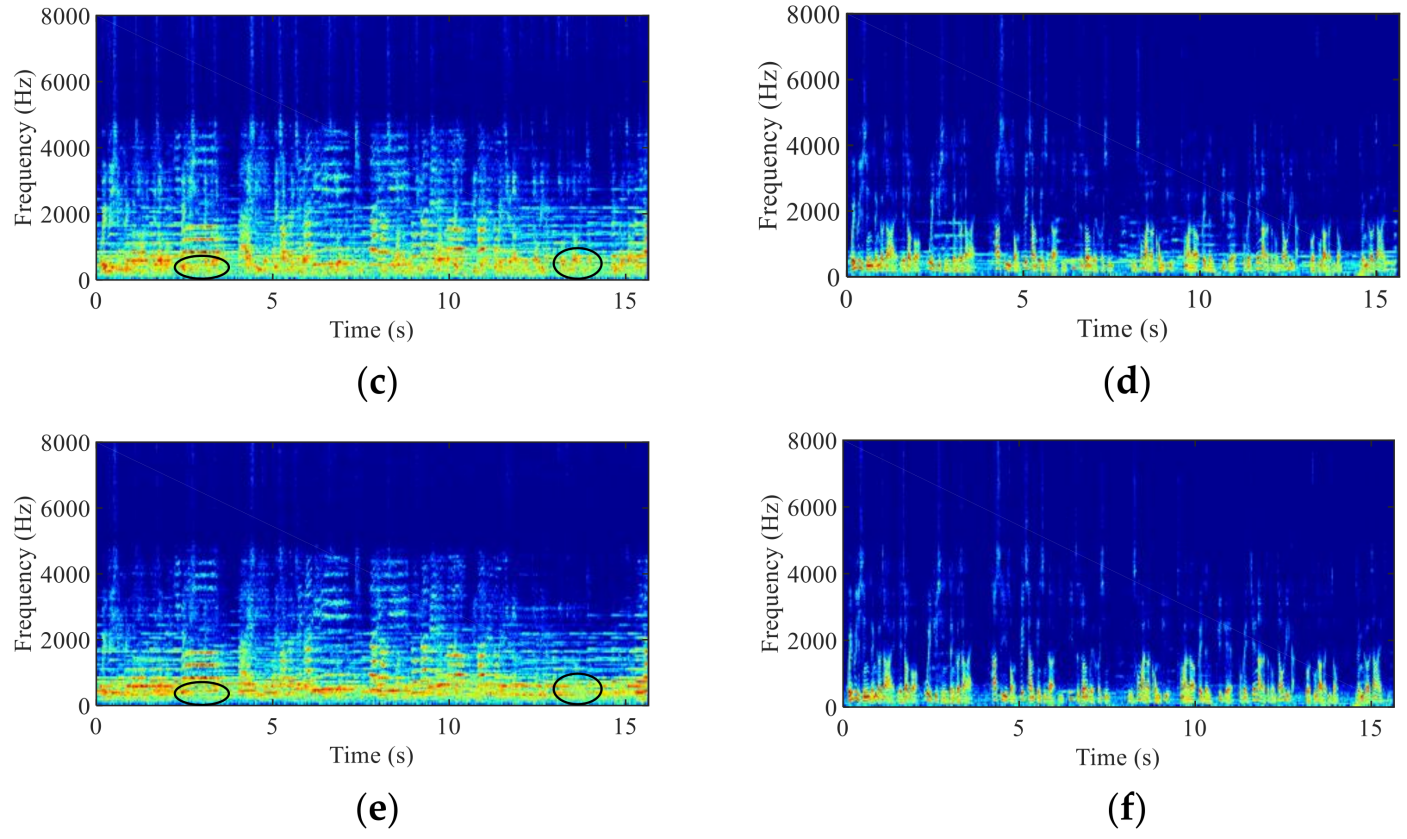
Speech spectrograms. (**a**) Clean AC speech; (**b**) Noisy signal at a single microphone; (**c**) ABM output of the conventional GSC; (**d**) Signal enhanced by the conventional GSC; (**e**) ABM output of the proposed BCA-GSC; (**f**) Signal enhanced by the proposed BCA-GSC.

The noisy AC speech signals were generated by corrupting the clean AC speech with noise under various SNRs (0, 5 and 15 dB). The BC speech signals and clean AC speech signals were recorded simultaneously in the absence of background noise (indoor, silent environment). The clean AC speech signals were at 0°. To demonstrate the robustness of the proposed BCA-GSC algorithm, four types of noise including directional noise and diffuse noise were used, and these noises were recorded indoors separately. Specifically, directional noise was obtained by a loudspeaker playing noise in a certain direction, and diffuse noise was obtained by simultaneously playing noises from four loudspeakers placed respectively in the four corners of the room. Figure 6 depicts the spectrograms of clean AC speech, noisy signal, ABM output of the conventional GSC, signal enhanced by the conventional GSC algorithm, ABM output of the proposed BCA-GSC, and signal enhanced by the proposed BCA-GSC algorithm in the case of the speech signal with 5 dB SNR and music noise at 90°. It can be seen that some harmonics of the desired signal remain in Figure 6c, while Figure 6e nearly does not contain the desired signal, such as the circled area. The residual noise in Figure 6f is also obviously less than that in Figure 6d. It means that the proposed BCA-GSC algorithm not only suppresses noise better, but also prevents the desired signal cancellation.

**Figure 7:**
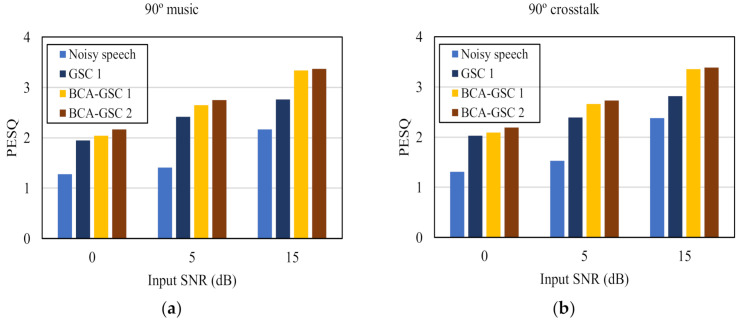

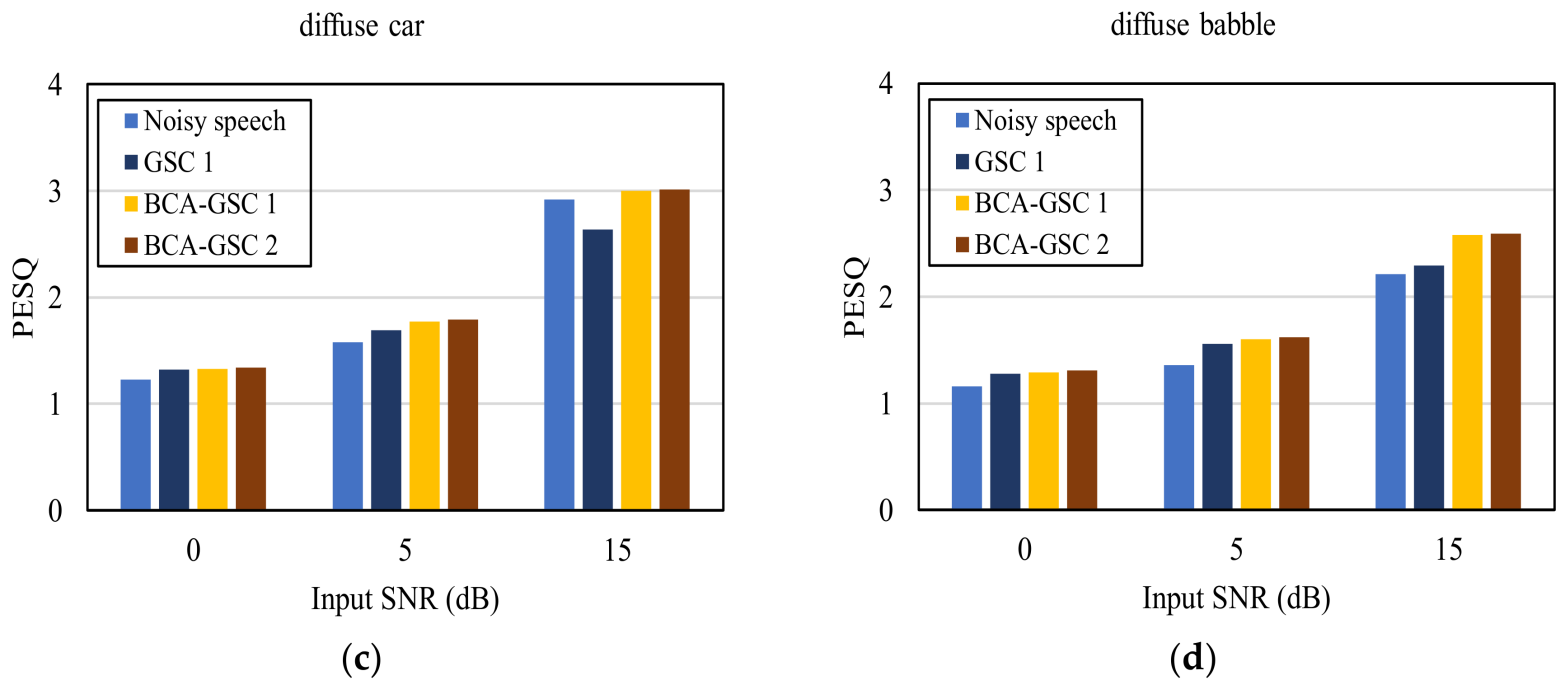
Quality in terms of PESQ of the objective test under different noise environments. (**a**) 90° music noise; (**b**) 90° crosstalk noise; (**c**) diffuse car noise; (**d**) diffuse babble noise.

To further verify the advantage of the proposed algorithm, the objective test perceptual evaluation of speech quality (PESQ) [29], which is highly correlated with subjective listening test, was conducted. Figure 7 shows the PESQ values at three background noise levels. GSC 1 denotes the conventional GSC algorithm [5], and BCA-GSC 1 and BCA-GSC 2 denote the BCA-GSC algorithm without iteration and one iterative BCA-GSC algorithm respectively. It can be seen that both BCA-GSC algorithms achieved an obvious improvement on PESQ scores in various noise environments, especially in the case of directional noise. Compared with the BCA-GSC algorithm, the conventional GSC algorithm improves PESQ scores less, and it occasionally leads to a drop in PESQ scores. In addition, the lower the SNR is, the more obvious the effect of iteration will be.

**Figure 8:**
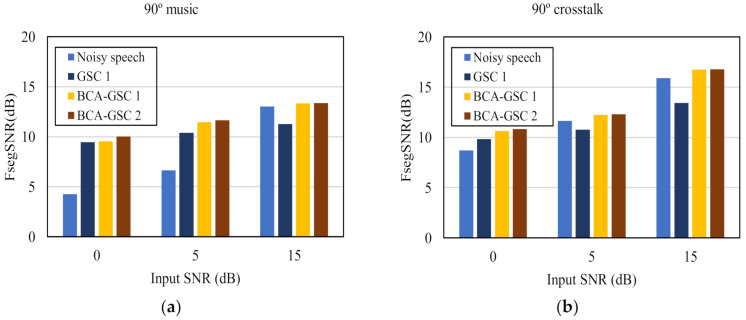

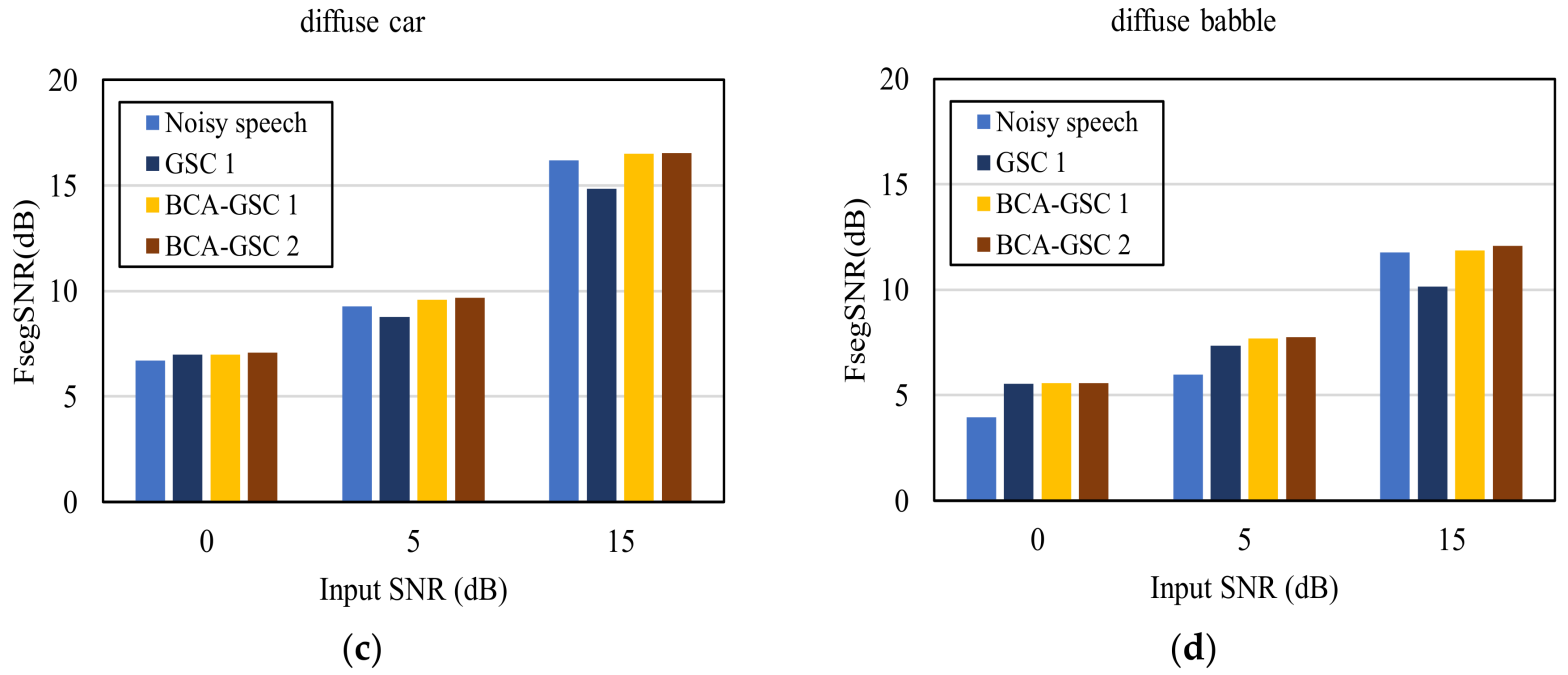
Quality in terms of FsegSNR of the objective test under different noise environments. (**a**) 90° music noise; (**b**) 90° crosstalk noise; (**c**) diffuse car noise; (**d**) diffuse babble noise.

The frequency domain segment SNR (FsegSNR) [30] measure was also conducted for evaluations, which has been shown relatively reliable for assessing speech quality. As shown in Figure 8, the proposed BCA-GSC algorithm improves the FsegSNR in each background noise situation. Likewise, iteration improves the performance of the BCA-GSC algorithm. The conventional GSC algorithm has a limited improvement and decreases the FsegSNR in high SNR case, which can be attributed to the inevitable elimination of the desired signal and the incomplete suppression of the noise.

PESQ and FsegSNR are objective measure of the speech quality. For evaluating speech intelligibility performance, short time objective intelligibility (STOI) measure was performed. Table 2 shows the scores of the STOI measure. A similar result can be observed. After being processed by the BCA-GSC algorithm, the STOI scores have been enhanced. Although the effect of iteration is insignificant, it brings no negative optimization. However, the conventional GSC algorithm often reduces the STOI scores, especially in high SNR conditions. This illustrates that the traditional GSC algorithm is not as good as the proposed BCA-GSC algorithm for desired signal protection.

## 5. Conclusions

In this paper, an improved robust GSC algorithm using two AC microphones and a BC sensor is proposed. The special characteristics of the BC sensor are exploited to obtain accurate VAD to control coefficients update of the ABM and ANC. The recursive least squares algorithm is employed to update the adaptive coefficients due to the efficiency in terms of convergence speed. The proposed BCA-GSC algorithm enjoys robustness under various background noise conditions, and provides a good noise suppression while protecting desired signal. The experiments demonstrated the proposed BCA-GSC algorithm improves both speech quality and intelligibility significantly, compared with the traditional GSC.

## Figures and Tables

**Table 1:** QRRLS implementation.

Initialization for node *k*:
R(0)=δI, with δ a small positive constant; u(0)=0 and w(0)=0 are null vectors.
**Update:**
Given R(n−1), u(n−1), w(n−1), the input x(n) and the desired signal d(n), we compute w(n) when the control factor is positive:
(i). The first update: $\left[\begin{array}{cc} R_{}^{\left(1\right)}(n) & u_{}^{\left(1\right)}(n)\\ 0^{T} & c_{}^{\left(1\right)}(n) \end{array}\right]=Q_{}^{\left(1\right)}(n)\left[\begin{array}{cc} \sqrt{\lambda (n)}R(n-1) & \sqrt{\lambda (n)}u(n-1)\\ x_{}^{T}(n) & d(n) \end{array}\right]$ The second update for *m* = (*n* mod *L*) +1: $\left[\begin{array}{cc} R(n) & u(n)\\ 0^{T} & c(n) \end{array}\right]=Q(n)\left[\begin{array}{cc} R_{}^{\left(1\right)}(n) & u_{}^{\left(1\right)}(n)\\ \sqrt{\kappa L}z_{m} & 0 \end{array}\right]$
where $Q_{}^{\left(1\right)}(n)$ and Q(n) are calculated by Givens rotation to obtain the left hand side of each equation above, *z_m_* is the *m*-th row of the identity matrix *I*.
(ii). $w(n)=R_{}^{-1}(n)u(n)$ (back-substitution).

**Table 2:** Intelligibility in terms of STOI (%) of the objective test.

Noise Type	SNR (dB)	Noisy	GSC 1	BCA-GSC 1	BCA-GSC 2
90^o^ music	0	72.05	90.58	91.13	92.11
	5	83.34	92.86	95.43	95.80
	15	95.19	94.12	97.66	97.68
90^o^ crosstalk	0	73.75	89.79	91.69	92.32
	5	83.69	92.35	95.44	95.61
	15	95.16	93.89	97.56	97.61
diffuse car	0	79.18	80.58	82.42	82.52
	5	87.80	87.05	89.72	89.75
	15	96.64	93.43	96.87	96.93
diffuse babble	0	62.19	71.61	71.81	72.02
	5	75.65	82.21	83.67	83.96
	15	93.70	92.14	95.47	95.51

## Data Availability

Not applicable.
